# Tumor-naïve ctDNA detection with deep learning-enhanced error suppression for sensitive mutation calling

**DOI:** 10.1186/s13073-026-01694-y

**Published:** 2026-06-23

**Authors:** Shaya Akbarinejad, Sarah Doppler, Jos de Graaf, Jelena Pistolic-Kraft, Valesca Bukur, Christian Albrecht, Nathalie Buchholz, Simge Özenoglu, Ugur Sahin, Jonas Ibn-Salem, David Weber

**Affiliations:** 1 TRON - Translational Oncology, University Medical Center of Johannes Gutenberg University Mainz gGmbH, Freiligrathstraße 12, Mainz, 55131 Germany; 2 Department of Biology, Johannes Gutenberg University Mainz, Mainz, Germany; 3 Johannes Gutenberg University Mainz, Mainz, Germany; 4 BioNTech SE, Mainz, Germany

**Keywords:** ctDNA mutation detection, Somatic variant calling, Tumor-naïve ctDNA assay, Deep learning, Machine learning, Liquid biopsy

## Abstract

**Background:**

Circulating tumor DNA (ctDNA) detection offers minimally invasive monitoring of cancer from blood samples. While tumor-informed approaches are sensitive, their clinical application is limited by cost, tissue availability, and turnaround time. In contrast, tumor-naïve assays offer faster turnaround times and broader applicability, but often sacrifice sensitivity.

**Methods:**

Here, we developed DEEPctMUT, a computational pipeline for tumor-naïve ctDNA detection that integrates unique molecular identifiers (UMIs) with three complementary error-polishing strategies: (1) machine learning-based sequencing error suppression, (2) deep learning-based background noise filtering (DeepES), and (3) removal of clonal hematopoietic and germline variants using patient matched PBMC. We designed an efficient sequencing panel and developed and validated DEEPctMUT using cell lines, spike-in samples, healthy, and CRC plasma samples.

**Results:**

DEEPctMUT accurately detected mutations down to 0.03% variant allele frequency (VAF), outperforming other tumor-naïve methods. In a head-to-head comparison, our pipeline identified pre-surgical CRC cases with 100% sensitivity, whereas the Roche Avenio Surveillance Kit achieved only 50% sensitivity. Furthermore, a panel-independent version of DEEPctMUT could improve the performance of Roche Avenio data without additional assay-specific training. We provide an extensive ground-truth dataset and make DEEPctMUT, including all trained models, available as an easy-to-use Nextflow pipeline.

**Conclusions:**

By using advanced computational error-polishing techniques, DEEPctMUT can substantially reduce technical artifacts, allowing our tumor-naïve method to achieve a sensitivity comparable to tumor-informed approaches.

**Supplementary Information:**

The online version contains supplementary material available at 10.1186/s13073-026-01694-y.

## Background

Circulating tumor DNA (ctDNA) from liquid biopsy has emerged as a powerful and minimally invasive biomarker to assess tumor burden in various research and clinical scenarios, such as early disease detection, treatment response, and minimal residual disease (MRD) monitoring for various cancers including colorectal cancer (CRC) [1]. Depending on tumor burden, the fraction of tumor derived circulating DNA can be very low within the pool of plasma derived cell free DNA (cfDNA), e.g. below 0.1% in early-stage disease [2]. Somatic mutations can differentiate ctDNA from cfDNA. However, ctDNA mutations occur at very low variant allele frequencies (VAF), making it challenging to differentiate true mutations from sequencing errors. For ctDNA analysis, there are two general detection approaches: tumor-informed and tumor-naïve. Tumor-informed approaches pre-determine a set of patient specific somatic mutations from a tumor biopsy or resection sample and monitor these mutations in plasma derived cfDNA either through deep sequencing with a patient-specific panel [3–7] or by shallow whole genome or exome sequencing [8, 9]. Because the target mutations are predefined, tumor-informed approaches can interrogate specific genomic loci, thereby improving the signal to noise ratio, enabling exceptionally sensitive detection, with limits reaching parts per million (PPM) levels. This makes them particularly powerful for MRD detection and post-surgical treatment monitoring [3, 6, 8–10]. However, tumor-informed assays require access to high-quality tumor tissue, which may not be available for all patients. Moreover, the development and validation of personalized panels is labor-intensive, time-consuming, and costly, which can delay critical clinical decisions. Additionally, tumor heterogeneity and clonal evolution can undermine the long-term relevance of the original tumor mutation profile [11]. 

In contrast, tumor-naïve approaches aim to detect somatic mutations directly without prior knowledge of the tumor’s mutations. Similar to tumor-informed methods, plasma derived cfDNA can be sequenced either via deep sequencing, in contrast to tumor-informed on a fixed panel [12–14], or also through whole-genome or exome sequencing [15, 16]. Tumor-naïve assays are more cost- and time-efficient, as they do not rely on tumor tissue and patient-specific panel design. However, the absence of a priori mutation information presents a significant challenge: distinguishing true mutations from sequencing artifacts becomes considerably more difficult. This limitation results in reduced sensitivity, particularly in samples with low ctDNA levels [17]. One strategy is to add unique molecular identifiers (UMIs) before amplification and sequencing to remove sequencing errors induced during PCR amplification by collapsing reads with same mapping position and same UMI into consensus reads [18–22]. However, other types of errors need to be addressed with data-driven and computational approaches. 

To polish errors in ctDNA sequencing, Newman et al. [23] proposed the integrated digital error suppression (iDES) algorithm, which fits a normal or Weibull distribution to each individual position and substitution on a previously sequenced cohort of healthy donor plasma samples. If the signal observed in a new sample is significantly higher than the position and substitution-specific fitted model, then a mutation is called. A key challenge for iDES, however, is limited training sample size. Especially for zero-inflated positions (those with no observed errors in the cohort), limited sample size can lead to insufficient evidence for robust error modelling across the entire panel. To mitigate this issue, Deng et al. [24] developed TNER, a Bayesian approach that uses the genomic context of mutations in form of tri-nucleotide-specific error rate to compensate for the lack of available data from healthy plasma, updates it with observed VAFs from healthy controls as evidence, and calculates a posterior error rate. This posterior probability can also be used to assess the significance of detected VAF. While effective, this Bayesian framework is difficult to extend to include additional genomic or experimental features, limiting its flexibility. Furthermore, both methods are inherently dependent on fixed sequencing panels, as their error models are fitted in a position-specific manner. As a result, applying these methods to a new panel requires sequencing of multiple healthy plasma samples to retrain a new position-specific error model, limiting their application to other panels designed for different tumor types. Another key limitation of relying solely on these error-suppression techniques in tumor-naïve settings is that not all significantly elevated VAFs correspond to somatic mutations from tumor derived ctDNA. Some positions exhibit high signal but with poor sequencing quality, while others may result from clonal hematopoiesis, a well-known confounding factor in plasma-derived cell-free DNA analysis [25–27].

A key limitation in training and evaluating error-polishing models for ctDNA analysis is the limited availability of high-quality ground truth data with known positive and negative mutations. While diluting deeply sequenced cell lines can be useful for replicating low-VAF mutations and thus modeling technical errors, this data fails to capture key biological features of true cfDNA, such as fragment size distributions. Spiked-in samples, for which mutation-bearing DNA fragments are mixed into cfDNA extracted from healthy donor plasma, were employed to offer a more biologically relevant model, as the healthy plasma background more closely reflects native cfDNA characteristics. Furthermore, plasma from tumor and healthy patients can serve as a ground truth, albeit with certain limitations. Mutations in matched tumor tissue samples can serve as positive instances for plasma mutation calls, although they might represent only an incomplete subset of all true mutations due to intra- and inter-tumor heterogeneity. Plasma from healthy donors can serve as a valuable negative control, as all mutation calls in such samples can be considered likely false positives. These aspects need to be considered carefully when choosing and creating training and evaluation data for computational ctDNA analysis approaches.

Here we assessed whether combining an optimized sequencing assay, advanced deep learning for error suppression, and machine learning on multiple mutational features can enhance the accuracy of ctDNA mutation detection towards a sensitivity comparable to tumor-informed approaches.

## Methods

### CRC panel design and evaluation

We downloaded somatic mutation calls per CRC patient as MAF file from the TCGA cohorts COAD and READ (Additional file 1: Table S1) [28]. Mutation coordinates were converted from hg38 to hg19 using UCSC liftOver tool. To allow design and evaluation of the panel on independent data, we split the 536 patients from the TCGA cohorts randomly into training data sets (*n* = 428, 80%) and test data set (*n* = 108, 20%). To increase the training data further, we downloaded mutation data from five additional CRC cohorts via the cBioPortal, which we filtered for chromosomes 1–19, X, Y and MT. The additional cohorts included 964 patients with 415,024 mutations (Additional file1: Table S1) [29–33].

We designed hybridization probes for all the mutations detected in the training dataset using the xGen™ Hyb Panel Design Tool on the IDT website (https://eu.idtdna.com/pages/tools/xgen-hyb-panel-design-tool) and obtained 291,111 probe regions consisting of 120 bp long genomic intervals. From these probe regions, we retained 279,059 probes with IDT design quality code “good”. We annotated the probe regions with all overlapping mutations from the training datasets. To optimize for maximum coverage of the patients, we ranked probes by the total number of CRC patients from the training dataset that have at least one mutation in the probe region. We considered only the top-ranking probes to achieve a cumulative panel size of 100 kb. The frequently mutated non-coding TERT promoter region chr5:1295209–1295329 (hg19) was manually added to the final panel [34]. See Additional file1: Table S2 for all loci included on the panel) 

To benchmark our CRC panel with other sequencing panels, we downloaded BED files of all panels used in the Sequencing Quality Control Phase II (SEQC2) consortium [35] (10.6084/m9.figshare.19128005.v2) and the Avenio panels “Targeted Panel”, “Expanded Panel”, and “Surveillance Panel” as provided by Roche. Using the test dataset, we assessed the number of covered mutations per patient and overall patient coverage. We provide the code for our panel design and evaluation as R package: https://github.com/TRON-Bioinformatics/paneldesign. 

### Sample collection

#### Healthy samples

All human samples were commercially purchased and collected under informed written consent with explicit permission for DNA testing. Healthy donor sample set (50 donors) were sourced from BioIVT (Westbury, NY, US): peripheral blood in cell-free DNA BCT tubes (Streck, La Vista, NE, US) for plasma preparation and cfDNA extraction/analysis; EDTA-anticoagulated peripheral whole blood as a normal germline control. 13 samples were used for training DeepES, 27 for validation of the whole pipeline and the rest 10 for comparison to Avenio.

#### CRC patient samples

CRC patient sample set (32 patients) were obtained from Indivumed (Hamburg, Germany): EDTA-anticoagulated peripheral whole blood derived plasma for ctDNA analysis; non-viable PBMCs as a normal germline control; FFPE tissue curls for inferring the tumor mutational profile (Additional file 1: Table S3). 22 samples were used for validation of the pipeline and 10 for comparison to Avenio.

#### Cell line and synthetic samples

DNA from 20 cell lines was either purchased from the Leibniz German Collection of Microorganisms and Cell Cultures Institute (DMSZ, Braunschweig, Germany) or extracted as indicated below. These samples were used to create dilution series to train and test RF model on.

Synthetic cfDNA Pan-cancer Reference Standards were purchased from Twist Bioscience (WT and 5% VAF; 3.0 µg per tube [San Francisco, CA, US]). These samples were used to validate the pipeline.

### DNA extraction and QC

Genomic DNA from cell lines was extracted from viably frozen cells (2–5 million cells were used per extraction) with the DNeasy Blood and Tissue Kit (Qiagen, Hilden, Germany).

Genomic DNA from FFPE tissue curls (four tissue curls à 10 μm per case and per tube, at least 10% tumor content, four tissue curls per case and per tube) was extracted with the Maxwell RSC DNA FFPE Kit (Promega, Walldorf, Germany). Genomic DNA from non-viable PBMC pellets sourced from CRC patient (approx. 2 million cells per case, stored at − 80 °C) was extracted with the DNeasy Blood & Tissue Kit (Qiagen, Hilden, Germany). Genomic DNA from healthy donor EDTA-anticoagulated peripheral whole blood (200 µL per reaction, stored at − 80 °C) was extracted with the QIAamp DNA Blood Mini Kit (Qiagen, Hilden, Germany).

For cell-free DNA analysis of CRC patients, we received frozen plasma (5–10 mL, stored at − 80 °C), which was derived from double-spun EDTA-anticoagulated peripheral blood. In the case of healthy donor samples, we received freshly collected peripheral blood stabilized in Cell-Free DNA BCT tubes (STRECK, La Vista, NE, US) at room temperature (60 mL). Within 36 h, we isolated the plasma from the STRECK vessels by double centrifugation at + 20 °C. For this, the plasma supernatant was separated by low-speed centrifugation at 1,600 x g for 20 min without a brake, followed by a second high speed centrifugation step at 16,000 x g for 10 min to remove any remaining cells and debris. Purified plasma (approx. 20–30 mL) was then stored in 1 mL aliquots in DNA LoBind tubes (Eppendorf, Hamburg, Germany) at − 80 °C until cell-free DNA extraction.

Cell-free DNA extraction from frozen plasma was carried out with the QIAamp MinElute ccfDNA Midi Kit (Qiagen, Hilden, Germany). A maximum of 10 mL plasma was prepared per purification column. Elution of cfDNA from the columns was done in 25 µL, and we reapplied the eluates a second time to increase the yield. The cfDNA size profile and gDNA contamination level was assessed via the Tapestation Cell-free DNA ScreenTape Analysis assay (Agilent Technologies, Santa Clara, CA, US). Samples were stored in DNA LoBind tubes (Eppendorf, Hamburg, Germany) at − 80 °C until library preparation. For CRC patient samples between 8 and 10 ml plasma and for healthy samples up to 30 ml of plasma was used (Additional file 1: Table S4).

DNA concentrations were measured with both the NanoDrop Spectrophotometer (except for cell-free DNA) and the Qubit™ 1X dsDNA HS assay (Thermo Fisher, Carlsbad, CA, US).

### xGen library preparation and hybrid capture enrichment with TRON CRC panel

For the hybrid capture experiments, we applied the xGen NGS library preparation workflow from Integrated DNA Technologies (Integrated DNA Technologies, Inc., Coralville, IA, USA) and based our assay on the xGen cfDNA & FFPE DNA Library Prep MC Kit (kit version 1, manual document version 2 from December 2021). We followed the manufacturer’s instructions with specified reagents as well as implemented some adaptations to the process as indicated throughout this section. A maximum of 16 samples were processed in parallel in one batch using PCR 8 well strips and multichannel pipets whenever appropriate.

To start library preparation, we used 10–50 ng (cfDNA) or 100 ng (tumor, normal, and cell line gDNA; synthetic cfDNA standard) of input material diluted in low TE buffer for the pre-capture library preparation. In the case of high molecular weight gDNA, the input was first sheared to approx. 150 bp sized fragments using the Covaris ML230 instrument (Covaris, Woburn, MA, USA) using the preinstalled standard 150 bp program for microTUBE-50 AFA Fiber V2 strips. Shearing efficacy was controlled by analyzing 1 µL of the sheared sample with the dsDNA HS chip on a Bioanalyzer (Agilent Technologies, Santa Clara, CA, US). In the case of low concentrated cfDNA samples, the volume was reduced to the library preparation start point target volume of 50 µL via SpeedVac for 20–30 min at 41 °C and 10 mbar (Christ, Osterode am Harz, Germany). Then, DNA ends were repaired, adapters containing UMIs were ligated, samples were barcoded with dual unique indexes, and equipped for Illumina sequencing by PCR amplification. The pre-capture amplification cycle number was adjusted according to the input material and quantity (< 20 ng: 10 PCR cycles, 20–25 ng: 9 PCR cycles, > 45 ng 8 PCR cycles).

During pre-capture library preparation, all mixing or resuspension steps were performed by vortexing instead of pipetting. Elution of the final pre-capture library after the last clean-up step was done in 60 µL and was extended to 10 min. Quality assessment of the pre-capture library size and concentration was carried out with the 1X dsDNA HS Qubit assay (Thermo Fisher, Carlsbad, CA, US) and the dsDNA High Sensitivity Bioanalyzer assay (Agilent Technologies, Santa Clara, CA, US). Typically, the total yield was > 3.0 µg. The average hands on time for the pre-capture library preparation was 6 h and could be comfortably completed within one day. Otherwise, appropriate stop points were indicated in the IDT protocol immediately after the pre-capture PCR amplification (before clean up). Samples were stored at − 20 °C in DNA LoBind tubes (Eppendorf, Hamburg, Germany) until further processing.

For the enrichment of regions of interest, we used the xGen Hybridization Capture of DNA libraries workflow (kit version 1; manual document version 5 from December 2021) with the associated reagents specified in the manual. As indicated, we had some adaptations to the protocol. For the hybridization, we purchased our panel design as a xGen Custom Hyb Panel-Accel oligo pool of 96 reactions, which had a concentration of 200 amole per probe per µL. From the pre-capture libraries, 500 ng was used as an input material for hybridization, and we followed the ‘Plate Protocol’ with a maximum of 16 parallel reactions per batch, e.g., two PCR 8 well strips (never a plate). The input material was dehydrated with a SpeedVac (Christ, Osterode am Harz, Germany) as described by IDT; however, this step was performed in DNA LoBind 1.5 mL reaction tubes (Eppendorf, Hamburg, Germany) that were additionally sealed with a perforated piece of Parafilm to prevent spilling or contamination. Following complete dehydration for 20–30 min at 41 °C and 10 mbar, the dried DNA was carefully resuspended in hybridization buffer and incubated for an additional 5 min for a total resuspension time of 10 min. Of note, the hybridization buffer contained only 1/10 of the suggested panel concentration (final concentration per reaction: 0.0667 pmol). The missing volume was compensated with nuclease-free water. After resuspension for 10 min, samples were transferred to polypropylene 0.2 mL thin-wall 8 strip PCR tubes with domed cap strips (Axygen, Union City, CA, USA), and hybridization was performed as indicated in the manufacturer’s instructions for 16–18 h. Subsequently, the capture was performed as described in the manual. To minimize prolonged sitting times of samples at room temperature during all the steps performed at 65 °C, we processed the samples strip by strip instead of taking both strips out of the cycler at the same time. Importantly, we ensured correct incubation timings of each strip throughout the whole heated washing procedure. After the capture washing steps, strepdavidin beads were resuspended by vortexing and not pipetting. Finally, post-capture libraries were further amplified as described (13 cycles) with the adaptation of a final amplification primer dilution of 1:60 instead of 1:24 (e.g., primer added: 0.5 µL instead of 1.25 µL) into the 30 µL amplification reaction mix. The final AMPure cleanup step was performed as described above for the pre-capture library preparation with vortex mixing instead of pipetting and a final elution time of 10 min. The final post-capture libraries were again assessed for quality with the 1X dsDNA HS Qubit assay (Thermo Fisher, Carlsbad, CA, US) and the dsDNA High Sensitivity Bioanalyzer assay or the NGS High Sensitivity Fragment Analyzer assay (Agilent Technologies, Santa Clara, CA, US). Concentrations of final libraries were typically well below 4.0 ng/µL. Higher concentrations usually indicated the presence of unspecific background DNA. Final capture libraries were stored in DNA LoBind 1.5 mL reaction tubes (Eppendorf, Hamburg, Germany) at − 20 °C until sequencing. A list of all prepared libraries is shown in Additional file 1: Table S5.

### AVENIO ctDNA analysis library preparation and hybrid capture enrichment

To benchmark our assay, we randomly selected ten plasma samples from CRC patient and ten plasma samples from healthy donors, of which we prepared NGS libraries with the AVENIO ctDNA Surveillance Analysis assay from Roche (Roche, Basel, CH). For each sample, an equal DNA input amount was used in both assays. For the AVENIO library preparations, the manufacturer’s instructions for the AVENIO ctDNA Analysis Kit with the Surveillance panel were followed (kit version V2, manual document version 1.6 from July 2023). A maximum of 16 samples was prepared in parallel in one batch using PCR 8 well strips and multichannel pipets whenever possible. Of note, we did not make use of the by default included cfDNA isolation module as the cell-free DNA was readily extracted via our own procedures as described in the respective section above.

Briefly, low concentrated cfDNA samples were concentrated via SpeedVac for 20–30 min at 41 °C and 10 mbar (Christ, Osterode am Harz, Germany) to the library preparation input volume of 50 µL. DNA was prepared for adapter ligation, and the ligation reaction was initiated and incubated for 16–18 h over night. Ligation adapters included one out of sixteen available single indexes to barcode each sample individually. Afterwards, the ligated pre-capture DNA was amplified via PCR with a default of 12 cycles, cleaned up, and stored in DNA LoBind tubes (Eppendorf, Hamburg, Germany) at − 20 °C until further use. Quality assessment of pre-capture library size and concentration was carried out with the 1X dsDNA HS Qubit assay (Thermo Fisher, Carlsbad, CA, US) and the dsDNA High Sensitivity Bioanalyzer assay (Agilent Technologies, Santa Clara, CA, US). The hands on time for the AVENIO pre-capture library preparation was typically only 4–5 h. However, the procedure had to be split into two days due to the overnight ligation. The hybrid capture was also performed exactly as stated in the Roche manual. The final post-capture library QC was performed using the 1X dsDNA HS Qubit assay (Thermo Fisher, Carlsbad, CA, US) and the dsDNA High Sensitivity Bioanalyzer assay (Agilent Technologies, Santa Clara, CA, US). Capture libraries were stored in DNA LoBind tubes (Eppendorf, Hamburg, Germany) at − 20 °C until sequencing.

### Illumina NovaSeq 6000 sequencing

Immediately before sequencing, sample molarities were re-quantified by measuring 2 µL of each library with the 1X dsDNA HS Qubit assay (Thermo Fisher, Carlsbad, CA, US). The library molarity was calculated with the recent concentration measurement and previously determined maximum peak size of each library.

For the xGen libraries, we created 0.9 nM library dilutions and used these to set up a 0.9 nM multiplexed pool that was subsequently denatured and prepared for sequencing on a NovaSeq 6000 SP (20 plex) or S1 (40 plex) flow cell (v. 1.5; 300 cycles) including 1.0% PhiX spike-in for quality assessment (Illumina, San Diego, CA, USA). This resulted in a final loading concentration of 180 pM. Libraries clustered at approximately 40–50 M clusters per sample resulting in 80–100 M paired-end reads and were sequenced in 159-8-8-159 read mode. Typically, the sequencing runs reached > 80% pass-filter clusters and Q30 > 95% values.

For the 20 AVENIO libraries, we applied the NovaSeq XP workflow for individual lane loading since Roche provides only 16 single barcodes, not sufficient for a 20 plex pool. We followed the Illumina XP workflow for the SP mode, created 0.6 nM library dilutions, and multiplexed 10 samples in one 0.6 nM pool. The pools (two pools of ten samples with partly overlapping indexes) were subsequently denatured and manually loaded into the two NovaSeq 6000 SP flow cell lanes individually (v. 1.5; 300 cycles). This resulted in a final loading concentration of 120 pM. As per manufacturer’s instructions for AVENIO libraries, we spiked-in 15% of PhiX to balance low complexity within the first four bases of each AVENIO library construct. Libraries clustered in a comparable manner to the xGen samples, with approximately 40–50 M clusters per sample. This resulted in 80–100 paired-end reads and were sequenced in 151-8-0-151 mode. Of note, Roche suggests to sequence AVENIO libraries at a smaller depth of only 25 M clusters per library. The resulting raw reads were then converted to FASTQ files and analyzed by the accompanying Roche Oncology Analysis Software via the locally installed Oncology Analysis Server. This closed end to end solution outputs a readily analyzed VAF report, which then served as the basis for our benchmarking of our analytical assay’s performance. Of note, since the capacity of the Roche Oncology Analysis Server is not compatible with the data amounts produced by a total of 400 M clusters or 25 M clusters per sample, the demultiplexing and the analysis had to be run multiple times on smaller data subsets to not exceed the server’s capacities. 

### DEEPctMUT pipeline for mutation calling in plasma sequencing

The DEEPctMUT pipeline is implemented in Nextflow [36] as reproducible end-to-end analysis workflow and available on GitHub (https://github.com/TRON-Bioinformatics/DEEPctMUT, version 0.1.0). Initially, paired-end reads in FASTQ format were aligned to the hg19 reference genome using BWA (version 0.7.17) with default parameters. Unique Molecular Identifier (UMI) sequences, each 8 bps long, were corrected using the CorrectUmis function from fgbio (version 1.3.0, https://github.com/fulcrumgenomics/fgbio). Next, reads with the same start position and identical UMI sequences were grouped into families using the GroupReadsByUmi function from fgbio. Consensus reads were then generated by collapsing all reads within a family via CallMolecularConsensusReads from fgbio. A minimum of three reads per family was required for consensus generation, and a base quality score of at least 30 was needed. Afterwards, overlapping reads are clipped by fgbio’s ClipBam function. Following these steps, any position exhibiting a mismatch relative to the reference genome was considered as candidate mutation. For positions with mismatches from multiple alleles, the variant with the highest VAF was selected. Candidate variants were then subjected to three post-processing error polishing steps: (1) Deep Error Suppression (DeepES), a deep learning model to filter mutations with non-significant VAF signal, (2) a Random Forest model (RF) to filter low-quality signal, and (3) an optional polishing based on matched PBMC samples to remove clonal hematopoiesis. These steps are explained below.

### DeepES error polishing for detecting abnormally high signal

To polish common position-specific errors, we developed a deep learning approach called DeepES. Intuitively, DeepES learns the expected background signal at each panel position for all possible substitutions. At application time, this background distribution is used to assess if an observed VAF signal is significantly higher than expected or not, and therefore, considered as mutation or error, respectively.

More specifically, DeepES learns the expected background VAF partially from the observed VAF in plasma samples from healthy donors. As healthy plasma samples should not contain ctDNA with true somatic mutations, these VAF signals are considered as errors during training. In total, the DeepES model uses 97 features (Additional file1: Table S6), which can be categorized into the following four groups:

1. Healthy donor VAF: A vector of observed VAFs for all four alleles (A, C, G, T) at all panel positions in 12 plasma samples from healthy donors.
2. Mutational signature features: The reference base, alternate base, 5’ and 3’ flanking bases (all one-hot encoded), as well as more granular features, such as the VAF of specific neighboring bases (e.g., VAF of 5’ base ‘A’, VAF of 5’ base ‘C’, etc.).
3. Quality features: mean and standard deviation of mapping quality, base quality, and variant position in read for multiples groups of reads, defined as reads supporting the reference allele, the alternate allele, each of the four nucleotides (A, C, G, T), as well as, reads mapped to the forward strand and reads mapped to the reverse strand (strand bias).
4. Fragmentation features: These include the mean and standard deviation of fragment lengths for reads supporting the reference and alternate alleles, as well as for each base (A, C, G, T) separately.

Features from group 1 are calculated from 12 healthy plasma samples. In contrast, features from groups 2, 3, and 4 are from the sample being tested for ctDNA. Therefore, only features from group 1 depend on the exact same sequencing panel used in training, while the other features from groups 2, 3 and 4 are panel independent and can be used more flexible, e.g. for other sequencing panels and genomic positions not seen in the training data. Therefore, we trained two models, DeepES that utilizes all features and panel-independent DeepES (PI DeepES) that utilizes only panel independent features from groups 2, 3 and 4. All features are normalized by min-max scaling.

All features were calculated for reads that were properly paired and had an insert size of less than 500 bp. Using these features, the model was trained to predict the mean and standard deviation of a normal distribution. This distribution characterizes the expected error VAF of a specific position and substitution within a given sample. Specifically, the model performs heteroscedastic linear regression, where—unlike standard linear regression—the standard deviation of the noise term is also modeled as a function of the input features. The model solves the following regression equation:

$$\begin{aligned}{E}_{ij}=&{f}_{\mu\:}\left(X;\theta\:\right)+{\epsilon}_{ij};\\&{\epsilon}_{ij}\sim\mathcal{N}\left(0,{f}_{\sigma\:}{\left(X;\theta\:\right)}^{2}\right)\end{aligned}$$

where $\:{E}_{ij}$ is the expected error VAF of position $\:i$ and substitution $\:j$, $\:{f}_{\mu\:}$, and $\:{f}_{\sigma\:}$ are the models predicting mean and the standard deviation of expected error, respectively. $\:X$ is the input features, $\:\theta\:$ is the model parameters, and $\epsilon_{i,j}$ is the random noise. The model solves the above equation by minimizing the gaussian negative log-likelihood loss function:

$$\begin{aligned}{\mathcal{L}}_{\mathcal{N}\mathcal{L}\mathcal{L}}\left({E}_{ij},f\left(\mu\:\right),f\left(\sigma\:\right)\right)=&\frac{1}{2}log\left(2\pi\:f{\left(\sigma\:\right)}^{2}\right)\\&+\frac{{\left({E}_{ij}-f\left(\mu\:\right)\right)}^{2}}{2f{\left(\sigma\:\right)}^{2}}\end{aligned}$$

We used neural networks to model $\:{f}_{\mu\:}$, and $\:{f}_{\sigma\:}$. A schematic representation of the model architecture is shown in Additional file 2: Fig. S1A. First, all features are normalized using a min-max scaler. The vector of 12 healthy plasma VAFs were processed through two layers of 1D convolutional neural networks (CNN), which serve as a parameter-efficient feature extractor to learn summary statistics. The remaining features were passed through two fully connected (dense) layers. These two branches were then merged and followed by two additional fully connected layers. Batch normalization was applied between each layer to improve training stability and convergence. The network has two outputs for $\:f\left(\mu\:\right)$ and $\:f\left(\sigma\:\right)$ resulting in predicting $\:{E}_{ij}$ as follows:

$$\:p\left({E}_{ij}|X;\theta\:\right)=\mathcal{N}\left({E}_{ij}|{f}_{\mu\:}\left(X;\theta\:\right),{f}_{\sigma\:}{\left(X;\theta\:\right)}^{2}\right)$$

A cohort of 13 plasma samples, which we deeply sequenced with our panel, was used to train this model. Substitutions with VAF > 1% were excluded. During each run of cross-validation, one sample was left out. For each position and nucleotide substitution, the model utilized the VAFs from 12 healthy samples and the additional sample-specific features (groups 2, 3, and 4) to predict the pre-UMI-collapsing expected error rate of the left-out sample. This cross-validation cycle was repeated until all 13 samples were in the left-out set once.

At application time, the network predicts $\:f\left(\mu\:\right)$ and $\:f\left(\sigma\:\right)$ for all candidate mutations based on the 97 features. The vector of 12 healthy plasma VAFs is pre-selected from the pool of 13 healthy plasma samples by prioritizing non-zero VAFs. Taking $\:f\left(\mu\:\right)$ and $\:f\left(\sigma\:\right)$ from the model, a one-sided z-score and a corresponding *p* value is calculated based on the observed VAF. *p* values calculated for all candidate mutations were subsequently corrected using the Benjamini-Hochberg method. If the corrected *p* values is significant (q-value ≤ 0.01), the substitution is flagged as a positive.

The model was implemented using TensorFlow version 2.11.0, and the min-max scaler was applied using scikit-learn version 1.3.2. Benjamini-Hochberg false discovery rate correction was performed using the statsmodels Python package (version 0.14.4).

### RF polishing for determining true mutations from false positives

While DeepES effectively identifies variants with statistically significant signals, a subset of these high-VAF mutations may still be attributed to technical artifacts, noise, or low-quality sequencing data in the analyzed sample. To distinguish true mutations from sequencing errors, we trained a Random Forest model on cell line data. As described in more details below, positive labels corresponded to mutations with a VAF > 10% in undiluted cell lines, while negative labels were mutations with a VAF < 0.3%. The RF model used the same features as DeepES, except for the 12 healthy sample VAFs. Additionally, under the “Mutational Signature Features” category, extra VAF features were added: the VAFs of the reference allele, alternate allele, and the VAFs of each individual base (A, C, G, and T). As in DeepES, features were normalized using min-max scaling. The full list of features is described in Additional file1: Table S7. To address class imbalance, we employed the UnderBagging technique [37, 38] in our RF model. In this approach, the majority class is under-sampled for each decision tree, while the minority class remains unchanged. The RF model is comprised of 100 decision trees, each with a maximum depth of 15, and was implemented using scikit-learn version 1.3.2.

### Matched PBMC polishing

This optional step employs matched PBMC data to further refine mutation calls and filter out potential artifacts. Mutations were discarded if their VAFs in the pre-deduplicated matched PBMC sample exceeded 1%, indicating a possible germline variant or clonal hematopoiesis event. Additionally, a one-sided binomial test was performed. The VAF observed in the pre-deduplicated PBMC sample served as the null hypothesis. If the number of reads supporting a mutation in the test sample was significantly higher (*p* value < = 0.05) than the expected number of supporting reads under the null hypothesis considering the coverage, the mutation was retained.

### Analyzing avenio samples

Avenio samples are analyzed by Avenio Surveillance kit v2 and the default variables of the Roche Oncology Software v2.1.0.

For the analysis of Avenio samples using panel-independent DeepES and the RF model, candidate mutations were derived from UMI-collapsed BAM files generated by the Avenio pipeline. As in DEEPctMUT, all positions exhibiting a mismatch were initially considered as candidate mutations. Subsequently, only those mutations overlapping with the predefined Avenio hotspot list were retained. DeepES and RF were applied using the same cut-off thresholds as DEEPctMUT. Minimum VAF argument was also set as 0.05%, the same as DEEPctMUT without PBMC.

### Tumor-normal variant calling

We also performed deep sequencing on tumor and matched PBMC samples from 32 CRC patients. UMI-deduplicated BAM files (as described in the previous section) from these samples were processed using Mutect2 [39] with default parameters as implemented in the TronFlow-Mutect (v1.4.0 https://github.com/TRON-Bioinformatics/tronflow-mutect2) to identify somatic variants, which served as tumor-confirmed mutations in our analysis.

### Cell lines ground truth data

To define the ground truth data, reads were mapped to the reference genome and UMI-collapsed as described above for DEEPctMUT. To prepare negative and positive sets for training and validation of the RF model, we used the information from the undiluted samples. Here, substitutions in panel positions with an observed VAF > 10% in at least one of the undiluted cell lines were designated as the positive set of true mutations (Additional file 1: Table S8). Mutations with 0.3% < VAF < 10% in at least one undiluted cell line were classified as uncertain and ignored from further analysis. All remaining observed very low frequency calls were assigned to the negative set.

To calculate expected VAF for the positive set in the 1:20 diluted sample, which is essentially a mix of the 20 cell lines without any further dilution, we divided the total number of mutant reads for each mutation by the total sequencing depth at that position across all 20 undiluted cell lines. The expected VAF for 1:100, 1:1000, and 1:10000 diluted cell lines were calculated by division with the additional dilution factors (5, 50 and 500). Calculating expected VAF for the negative set is not trivial, because dilutions have, in principle, no effect on background noise signals. Therefore, we defined the expected VAF for the negative set as the VAF observed in an independent diluted cell line sample that was excluded from the RF model’s training and cross-validation (Additional file 1: Table S5). Using the signal from a replicate, allows a more realistic measure of negative signal in diluted cell lines that is decoupled of run-specific noises in samples used in training or validation.

For diluted cell lines, after mapping and UMI collapsing, candidate mutations were defined as all positions exhibiting a mismatch to the reference genome. Mutations overlapping the predefined positive and negative sets were then used for training and validation of the RF model.

### TWIST samples analysis

For ground truth definition reads were mapped to the reference genome and UMI-collapsed as described above for DEEPctMUT. As positive set we utilize the 15 spiked-in mutations in the TWIST sample that overlap with the TRON CRC panel. These have predefined VAFs according to the manufacturer. The expected VAFs for the 1:10, 1:50, 1:500, and 1:5000 dilution were calculated by dividing these known VAFs by their respective dilution factors. The negative set was defined using the spike-in free background sample also provided by the manufacturer. Here we considered any mutation call with a VAF < 10% as negative and used the observed VAF as expected VAF in the negative set (Additional file 1: Table S5). 

## Results

### The TRON CRC ctDNA detection panel enriches hotspot mutations and minimizes off target coverage

**Fig. 1:**
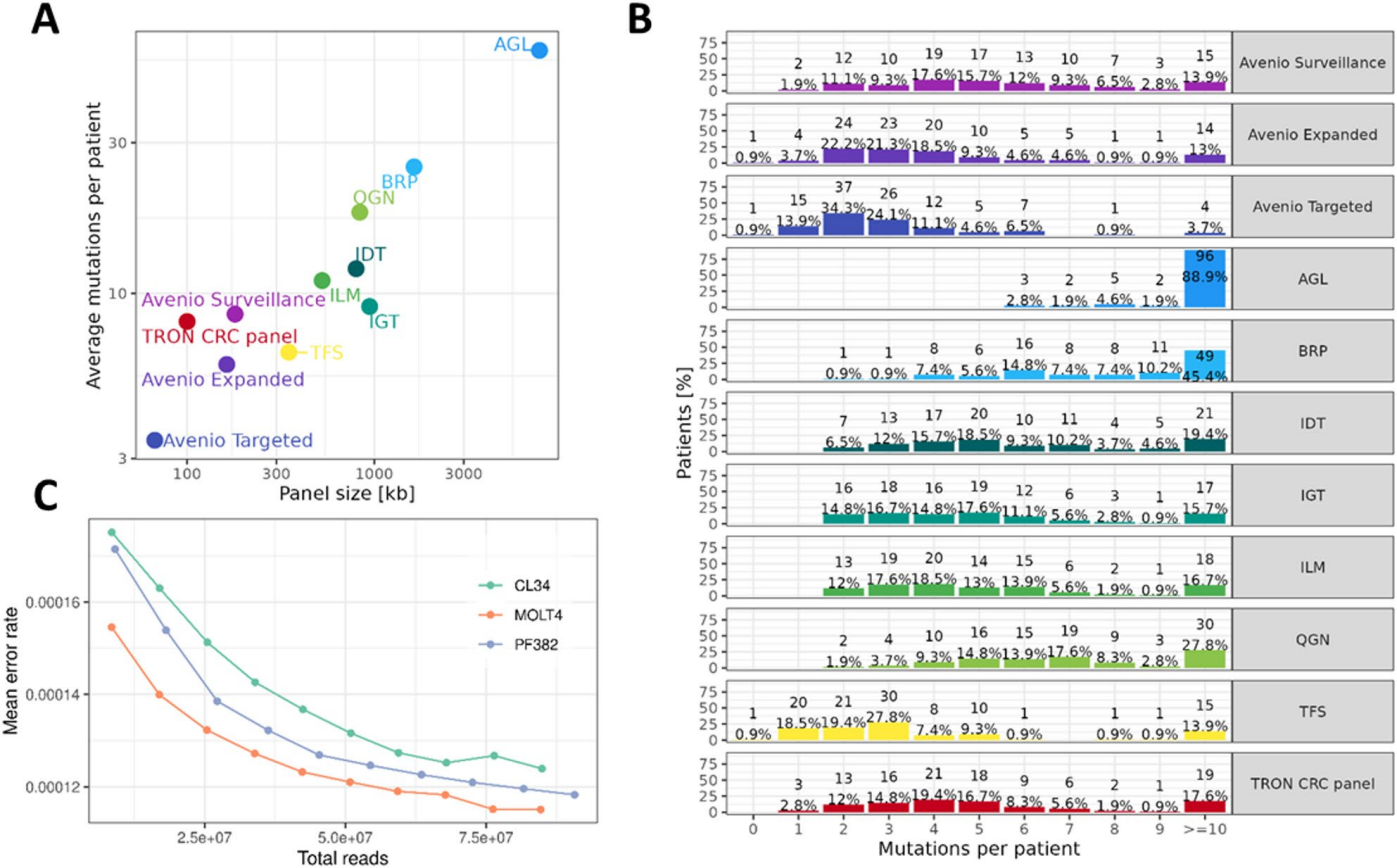
Limitations of ultra deep sequencing for UMI based sequencing error correction on a custom CRC capture panel. **A** Shown is the average number of mutations covered by each panel according to respective panel size in kb as observed in the test subset of CRC patients (*n* = 108), not used for TRON CRC panel design. Other panel include Agilent Custom Comprehensive Cancer Panel v2 (AGL), Burning Rock DX OncoScreen Plus (BRP), Integrated DNA Technologies xGen Pan-Cancer Panel (IDT), iGeneTech AIOnco-seq (IGT), Illumina TruSight Tumor 170 (ILM), QIAGEN Comprehensive cancer panel (QGN), Roche SeqCap EZ Choice custom PHC Panel (ROC), and Thermo Fisher Oncomine Comprehensive Assay v3 (TFS). **B** Histogram showing how patients of the CRC test dataset (*n* = 108) distribute across different coverage levels ranging from not covered = 0 mutations to > = 10 mutations. **C** Estimated mean error rate by raw sequencing depths via down-sampling of sequencing reads for the three cell lines CL34, MOLT4, and PF382

To establish our approach, we developed a custom hybridization capture panel to sequence hotspot mutations in colorectal cancer (CRC) patients. CRC is known to have well detectable ctDNA levels and is therefore well suited, to establish our approach of tumor-uninformed mutation detection [40, 41]. We designed the CRC panel using somatic mutations from public available CRC cohorts from TCGA and cBioPortal (Additional file 1: Table S1). We split the data by patients into a panel-development dataset (*n* = 1392) and a test dataset (*n* = 108; Additional file 2: Fig. S1B). A custom CRC capture panel was designed according to 120 bp target regions optimized for the number of CRC patients covered with somatic mutations in the panel-development dataset to enrich for highly recurrent mutations. In total 834 hotspot mutation regions were included in a panel with 100 kb total size (Additional file 1: Table S2). When comparing different sequencing panels using the test dataset of CRC patients (*n* = 108), we observed that the average number of covered mutations increases almost linearly with panel size (Fig. 1A). Our CRC panel with an average of 8.14 covered mutations per patient provides the best ratio between panel size and mutation coverage (Fig. 1A and Additional file 2: Fig. S1C). It covers all patients of the test cohort with at least one mutation. Three patients (2.8%) are covered by only one mutation, while the majority (*n* = 105, 97.2%) are covered by multiple mutations. Thus, our panel provides similar coverage as the commercial Roche Avenio Surveillance panel, which is double in size (Fig. 1B). We used the IDT xGen hybrid capture cfDNA & FFPE library preparation system, which was previously shown to enhance ligation efficiency enabling library generation for low input amounts (> 1 ng) [42]. We tested three capture probe concentrations and observed stable mean target sequencing coverage and on target rates (81–84%) across 100-fold dilution range (0.67 pmol – 0.0067 pmol; Additional file 2: Fig. S1D). The target rate was slightly improved at 1:10 dilution (0.067 pmol), which we used for all further experiments. We selected a set of 20 cancer cell lines based on the number of known mutations overlapping with our custom CRC panel. Systematically reduced coverage was observed for only 6 out of 834 target regions, which all account for relatively rare CRC mutations (< 2% in CRC test dataset) and are, therefore, less critical for the overall performance (Additional file 2: Fig. S1E and F).

We went on to investigate how sequencing depth affects coverage after single strand collapsing as well as UMI family sizes, the number of sequencing reads sharing the same UMI and mapping position. For that, we consider UMI families with at least three raw reads as informative for UMI error correction. At a coverage of 20.000 X raw reads (according to the manufacturer’s recommendation), UMI families with less than three reads still exist (20–30%), which would be indicative that deeper sequencing could potentially further boost UMI error correction ( Additional file 2: Fig. S1G). However, when we estimated raw sequencing error after UMI correction, we observed that already at lower sequencing depths a plateau was reached (Fig. 1C), indicating that UMI error correction cannot be further improved by further increasing sequencing depth.

### A random forest model improves prediction accuracy for low frequency mutations

**Fig. 2:**
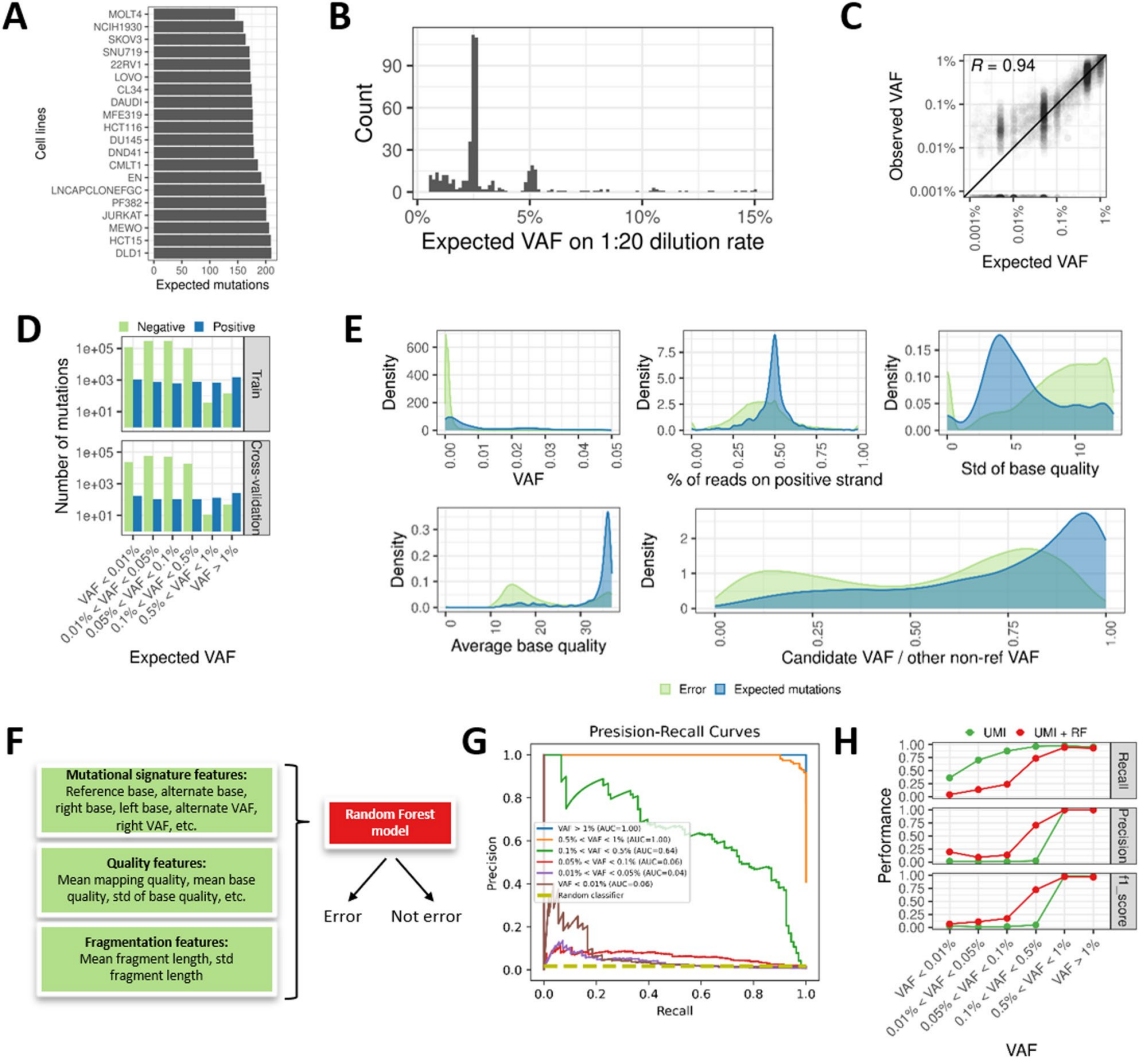
A random forest can distinguish sequencing artifacts from mutations and improve over UMI collapsing. **A** Number of true positive mutations (defined as VAF > 10%) per cell line on the TRON CRC panel for 20 selected cell lines. **B** Expected VAF distribution of true positive mutations according to 1:20 dilution. **C** Observed VAF after sequencing for all dilutions (1:20, 1:100, 1:1000 and 1:10000) is shown against the calculated expected VAF; R indicates the Pearson coefficient. **D** The number of true positive mutations (blue) and true negative positions (green) according to different observed VAF ranges for train and validation datasets. **E** Density distribution for five predictive mutation features for expected mutation (blue) and error positions (green). **F** Schematic diagram of feature families used in the RF model (for a full list see Additional file 1: Table S7). **G** Precision-recall curves showing the performance of the Random Forest model in validation data for different VAF ranges. Area under the curve (AUC) is indicated in the inlay. **H** Mutation detection performance (recall, precision, and f1) for UMI correction (green) and UMI correction with RF model (red) across different VAF ranges

To learn patterns of technical sequencing errors, we generated ground truth data of mutations across a wide VAF range by diluting extracted DNA from 20 cell lines. From the sequencing data of pure cell line DNA, we categorized panel positions that deviate from the human reference sequence as positive (VAF > 10%), uncertain (0.3% − 10%), and negative (VAF < 0.3%) sets. The lower VAF threshold of 0.3% was selected because we observed 99.5% of random errors fell below this value (Additional file 2: Fig. S2A). Using this approach, we confidently assigned between 146 and 211 true positive mutations for each cell line (Fig. 2A and Additional file 2: Fig. S2B). We selected the NCIH1930 cell line as background for DNA dilution because it exhibited the fewest overlapping positive mutations with other cell lines, resulting in 51 unique positive mutations after dilution. We applied four dilution levels (1:20, 1:100, 1:1000, and 1:10000) and sequenced each sample in three replicates, resulting in a total of 6204 known positive mutation in the ground-truth set with varying VAF levels. For example, expected VAF for the 1:20 dilution starts from 0.5% analyzed the diluted samples after sequencing and as expected, the actual observed VAF on UMI collapsed reads highly correlated with the expected VAF (Pearson correlation coefficient *R* = 0.94, Fig. 2C). However, this correlation declined for lower VAF ranges and could not be observed for very low VAF mutations (< 0.01%, Additional file 2: Fig. S2C). While the number of detectable mutations across different VAF ranges is relatively uniform (1227–1788, Fig. 2D), especially at very low VAF less are represented by UMI collapsed reads (*n* = 463 for VAF < 0.01%, Additional file 2: Fig. S2D). Despite this lack of read support, we still considered the complete set of all 517 true positive mutations across the entire VAF range. We split the dataset into training and validation sets by using mutations on chromosomes 1 and 2 for validation, and all others for training (Fig. 2D).

To improve mutation detection accuracy beyond UMI error correction, we analyzed 99 mutational and sequence quality features for distinguishing positive from negative candidate mutations and trained a random forest (RF) model to classify these (Fig. 2E and F). The most important features were VAF, average and standard deviation of base qualities of reads supporting a variant, fraction of reads supporting a candidate variant to reads supporting all other non-reference alleles, and strand bias ( Additional file 2: Fig. S2E). We assessed the performance of the RF model using cross-validation on the validation dataset for different VAF ranges, resulting in AUC of 1 for VAF > 0.5% and an AUC > 0.64 for VAF > 0.1%. As expected, the AUC decreases with lower VAF ranges, with only 0.06 AUC for very low frequency mutations (VAF < 0.01%, Fig. 2G). When analyzing the added benefit of RF model performance over UMI error correction, we observed an improved F1 score across all VAF ranges, except for the lowest range (VAF < 0.01%), with most notable improvement in the 0.1% − 0.5% VAF range, where the F1 score increased from 0.02 with UMI alone to 0.75 when the RF model was applied (Fig. 2H). Performance across three different replicates, with associated confidence intervals, is illustrated in Additional file 2: Fig. S2F. This indicates a substantial benefit of combining UMI-based sequencing with the RF model and an oval improved variant detection performance for low VAF mutations.

### DeepES improves error suppression over existing methods

The RF model can detect low-quality substitutions and polish common error patterns. However, it fails to account for specific positions with substantially higher local error rates. We aimed to learn position and substitution specific errors from healthy donor plasma samples, where no tumor-associated somatic mutations are expected. We sequenced in total 50 healthy plasma samples of which we used 13 for training. These reflect the fragment size distribution of cfDNA, which helps to prevent overfitting to different levels of genomic DNA contamination (Additional file 2: Fig. S3A). 

**Fig. 3:**
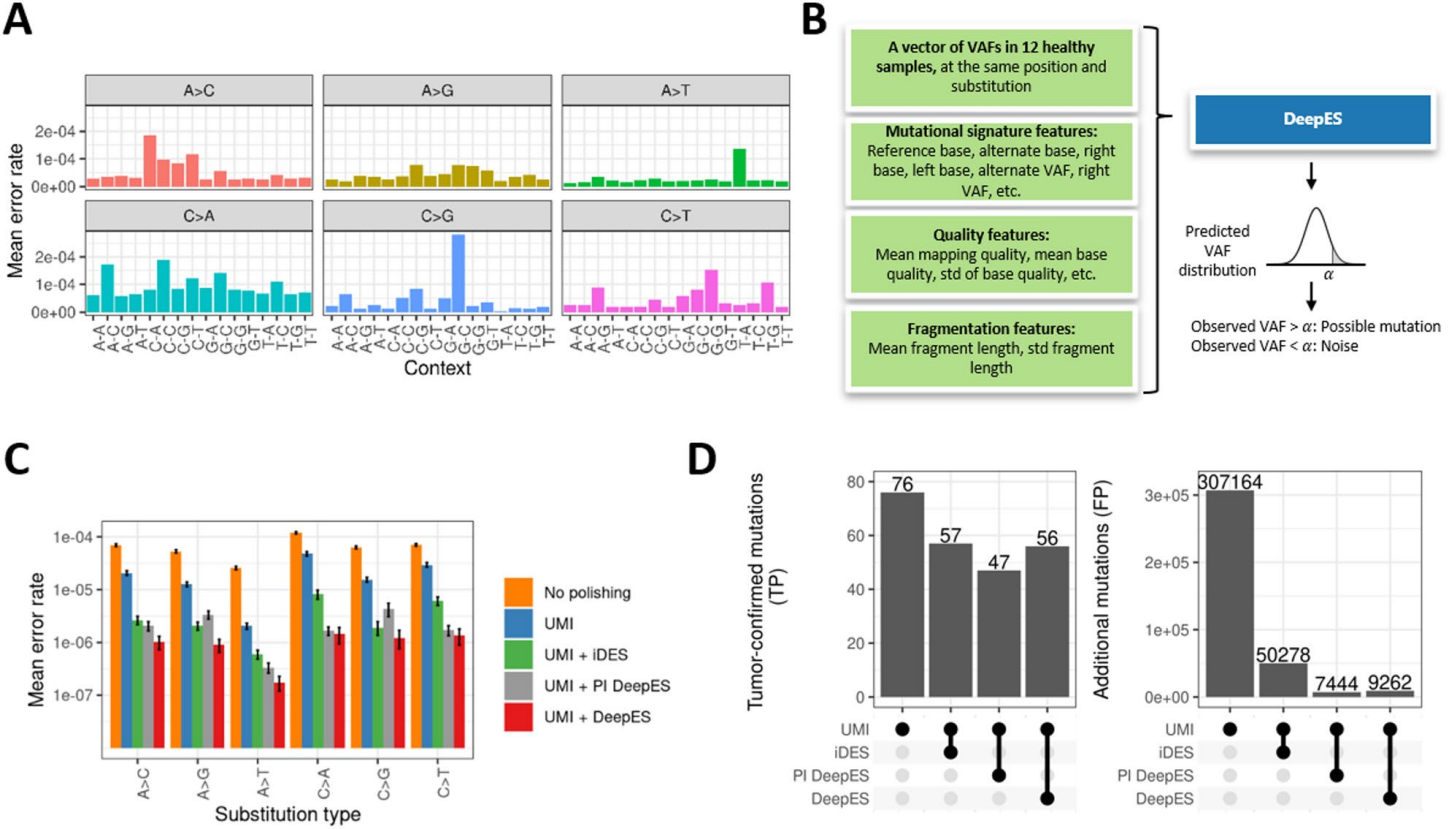
DeepES can improve prediction accuracy over iDES. **A** Trinucleotide context provides us with insightful information about error rate. These features are missing in iDES. **B** A schematic describing the DeepES model. **C** Tested on 27 healthy plasma samples, which were not used in training, UMI + DeepES, and UMI + Panel-Independent DeepES (PI DeepES) polishing has a lower error rate across all substitution types than UMI + iDES. Error bars show 95% confidence interval over 27 healthy plasma samples. **D** Comparing mutation calls between iDES, DeepES, and PI DeepES in 22 CRC plasma samples. Tumor confirmed mutations (true positive) are shown left, while additional mutations (a proxy for false positive) are shown on the right

Consistent with previous studies, different substitutions and tri-nucleotide contexts exhibited varying error rates (Fig. 3A and Additional file 2: Fig. S3B) [43, 44]. We investigated an expanded set of features —i.e. the ones used in the RF model— are informative to predict the observed error rates for a given position, such as “Percentage of reads on positive strand”, “Average template length of reads”, and “Average location of variant reads” (Additional file 2: Fig. S3C). To leverage tri-nucleotide context with these additional features for improved error polishing, we formulated the problem as a deep learning task. Our model, termed Deep Error Suppression (DeepES) uses four groups of features to predict the observed error VAF distribution, from which mutations in new samples can be called as positions with elevated VAF (Fig. 3B, Additional file 2: Fig. S1A, and Additional file 1: Table S6). The features include a vector of 12 VAFs of the same position and substitution in a cohort of healthy plasma samples (*n* = 13), and three other feature groups from the RF model. We trained a neural network in leave-one-out on all 13 healthy samples as a heteroscedastic regression task that predicts the mean and standard deviation of a normal distribution. From this, the error VAF from a left-out sample could be drawn (details in Methods). During application, only events that have a significantly (adjusted *p* < 0.01) higher VAF compared to the observed distribution in healthy samples are reported as candidate mutations (details in Methods). We trained an additional variation of the model without the VAF features from the healthy plasma samples. As this model does not depend on sequencing data of healthy donors from the same panel or any panel specific position data, we call this more flexible version Panel-Independent DeepES (PI DeepES).

For comparison, we trained iDES on the same cohort as DeepES and assessed performances of all approaches on 27 independent healthy plasma samples. All approaches (iDES, DeepES, and PI DeepES) improve error rates compared to using UMIs alone (Fig. 3C). However, DeepES consistently outperforms iDES in all substitution types and in most tri-nucleotide contexts (Additional file 2: Fig. S3D). Overall, DeepES reduces the error rate by factor 4.7 compared to iDES, and by 26.6-fold compared to using UMIs alone. Remarkably, PI DeepES overall also outperformed iDES, achieving a 2.8-fold reduction in error rate, although it exhibited inferior performance relative to iDES for A > G and C > G substitution errors. This data indicates that DeepES improves error suppression over existing methods and PI DeepES can generalize to panel-independent sequencing data.

To evaluate whether the improved error rate of DeepES compromises sensitivity, we sequenced 22 pre-surgery plasma samples from 21 CRC patients (two sample sets were derived from primary and relapsed CRC of the same patient). We also sequenced matched tumor and PBMC samples to identify somatic mutations (see Methods and Additional file 1: Table S9), allowing us to assess sensitivity in plasma by measuring how many tumor-confirmed mutations are detected. Additionally, mutations detected in plasma that are not found in the tumor serve as a proxy for false positives. While some of these tumor-unconfirmed mutations may represent subclonal or metastatic mutations not captured in the tumor sample, the majority are likely sequencing errors. Here, we observed that error suppression substantially improves specificity (Fig. 3D). iDES reduced the number of tumor-unconfirmed mutations by nearly an order of magnitude while maintaining 75% sensitivity (57/76). DeepES further reduced false positives by nearly another order of magnitude, while preserving comparable sensitivity (74%, 56/76). Thus, DeepES misses only one tumor-confirmed mutation over iDES, but had a 5-fold lower number of likely false positive mutation calls. PI DeepES also substantially reduced the number of additional mutations while still detecting 61% of tumor-confirmed mutations (47/76), highlighting its strong error suppression capability albeit a reduction in sensitivity.

### DeepES error polishing synergizes with RF error classifier and matched PBMC for ctDNA prediction

**Fig. 4:**
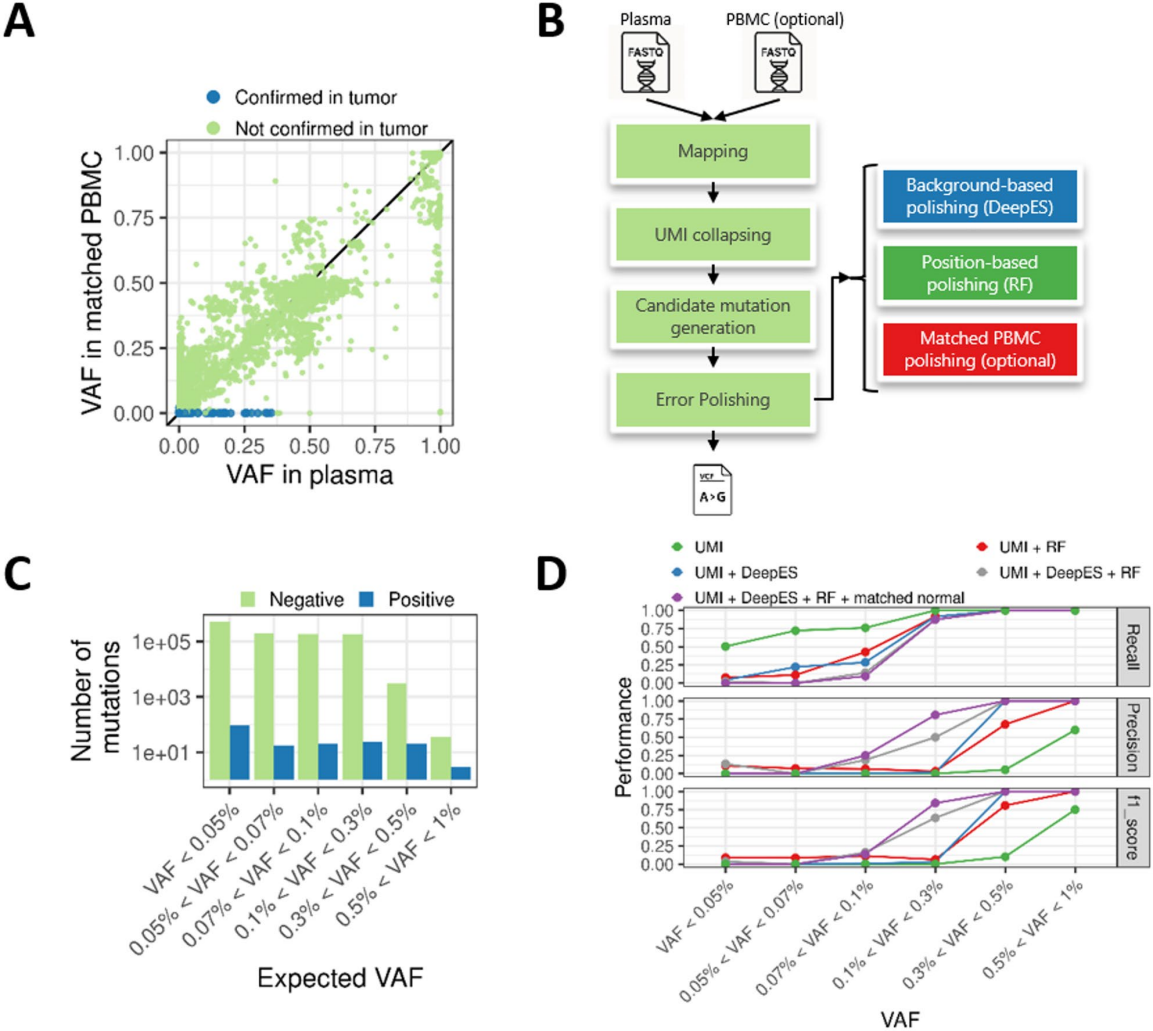
DeepES and RF classifier synergize for TWIST artificial ctDNA mutation detection. **A** Observed VAF in plasma cfDNA and matched PBMC DNA for tumor confirmed (blue) and not confirmed (green) mutations predicted in plasma cfDNA. **B** A schematic showing the steps of DEEPctMUT computational pipeline which includes: mapping to reference genome, UMI collapsing, candidate mutation generation, and three steps of error polishing. **C** The counts of true positive mutations (blue) and true negative positions (green) are shown for different observed VAF ranges in Twist spiked-in samples*. ***D** Recall, precision, and f1- score are shown for UMI (green), UMI + DeepES (blue), UMI + RF (red), UMI + DeepES + RF (grey), and UMI + DeepES + RF + matched PBMC (pink) across different VAF ranges

While the RF and DeepES models can effectively filter sequencing and background errors, they cannot distinguish whether high VAFs originate from somatic mutations, germline mutations or clonal hematopoiesis. As previously reported [45], we observe that tumor-confirmed mutations exhibit high VAF in plasma but low VAF in matched PBMC (Fig. 4A). Accordingly, we filtered out mutation candidates with VAF > 1% in matched PBMC. Additionally, we applied a binomial test to exclude mutations where the VAF in plasma was not significantly higher than in matched PBMCs (*p* value threshold = 0.05; see Methods for details). 

Finally, we combined all three polishing steps in the DEEPctMUT computational pipeline that calls mutation candidates from plasma sequencing data and employs multiple steps to suppress sequencing errors and to filter false positive mutation calls (Fig. 4B). First, we map reads to the human reference and group them based on UMI tags and mapping positions. Next, we discard groups with less than three reads as uninformative and collapsed others into consensus reads. Then, we inferred candidate mutations from the pileup data of the consensus reads and applied three polishing steps to retain only high-confidence mutations: (1) technical error classification using the RF model; (2) background polishing using a deep learning model (DeepES), which models and filters common position and substitution specific errors, and optionally, (3) an additional matched PBMC filtering step to filter out germline and clonal hematopoiesis mutations.

To test the full pipeline, we utilized highly controlled reference samples with artificial spike-in fragments that resemble the features of ctDNA (TWIST bioscience). Our CRC panel targets 15 of the included mutations that range from 3% to 6% VAF in the undiluted reference sample and could all be detected post-sequencing. We prepared four serial dilutions of the sample (1:10, 1:50, 1:500, 1:5000), each sequenced in three replicates, resulting in a total of 180 mutations instances. Other positions are considered as negatives (Fig. 4C, more details in Methods). Similar to the cell lines, we observed a correlation between observed and expected VAF according to the dilution (Additional file 2: Fig. S4A).

Using UMI + DeepES or UMI + RF, we observed high prediction accuracy at VAFs higher than (> 0.3%), but precision dramatically drops at lower VAFs (Fig. 4D). However, we observed complementary improvements using DeepES, the RF classifier, as well as the matched PBMC in the VAF range below down to 0.07%. Notably while precision is dramatically improved, sensitivity remains high. The most striking result was the improvement in F1 score for mutations down to 0.1% VAF, reaching an F1 score of 0.84. Performance across three independent replicates, including error bars, is detailed in Additional file 2: Fig. S4B. In this dataset, given the extreme class imbalance between positive and negative sets—particularly at lower VAF ranges—even a marginal number of false positives can lower precision and the overall F1 score.

### DEEPctMUT detection pipeline is highly sensitive even in early-stage CRC samples

**Fig. 5:**
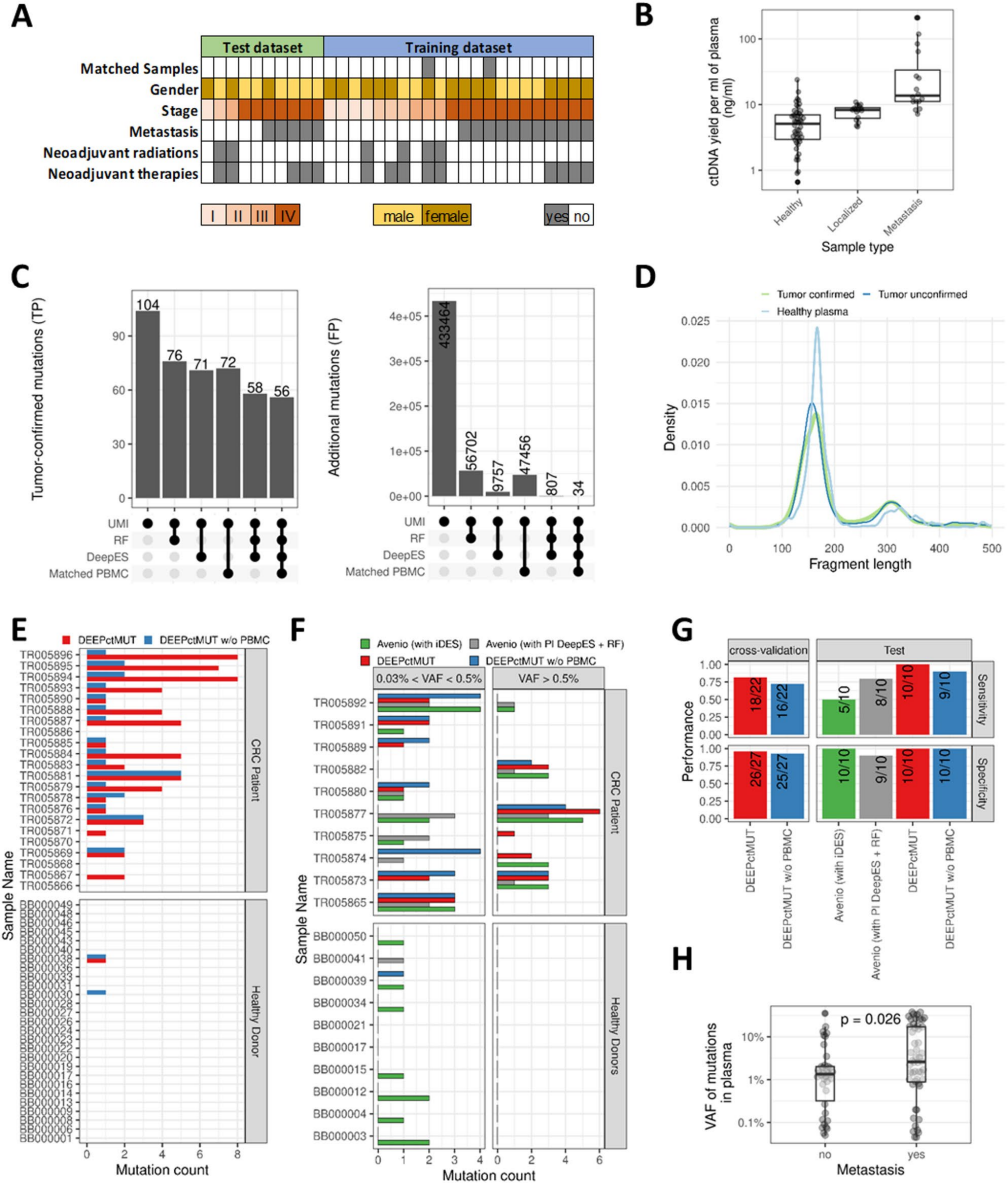
Our combined approach can accurately predict ctDNA in CRC patients. **A** Characteristics of 32 preoperative plasma samples derived from 31 CRC patients. For more details, see Additional file 1: Table S3. **B** cfDNA levels in ng/ml blood for healthy and CRC preoperative samples from primary and metastasized patients. **C** Comparison of mutation calls when applying different stages of the pipeline across 32 CRC patients. The left plot shows the number tumor- confirmed mutations, and the right plot shows tumor- unconfirmed mutations (a proxy for false positive) **D** Fragment length distribution of tumor confirmed, tumor unconfirmed, and healthy control mutations in plasma. **E** Called mutations of DEEPctMUT and DEEPctMUT without PBMC on a cohort of 22 CRC patients and 27 healthy controls. **F** Called mutations of DEEPctMUT, DEEPctMUT without PBMC, Avenio, and Avenio BAM files analyzed by PI DeepES and RF on an independent cohort of 10 CRC patients and 10 healthy controls according to two VAF ranges. **G** Patient-level performance of the DEEPctMUT, DEEPctMUT without PBMC, Avenio, and Avenio analyzed by PI DeepES and RF on 10 CRC patients and 10 healthy controls. For Avenio (with iDES), a VAF threshold of > 0.5% was applied. For all other pipelines, VAF > 0.03% was applied. **H** VAF with DEEPctMUT of mutations in metastatic and localized tumor samples

To evaluate the sensitivity and specificity of the entire pipeline, we used a cohort consisting of 32 preoperative plasma sample sets from 31 CRC patients, each with matched PBMC and tumor samples, as well as 50 plasma samples from healthy donors. Of the healthy plasma samples, 13 were used to train DeepES. A subset of 22 CRC and 27 healthy samples was used in cross-validation to specify the minimum VAF required for a mutation to be considered informative. The remaining 10 CRC and 10 healthy plasma samples were reserved as an independent test set to assess performance and compare it with the commercial Roche Avenio platform. We sampled a broad range from early to late stage tumor samples, with half having localized tumors at stage I to IV B and the other half having relapses with stage IV metastases in the liver, lung, or adrenal glands (Fig. 5A and Additional file 1: Table S3). As expected, total cfDNA concentration levels increased from healthy controls towards primary and metastatic CRC samples (Fig. 5B).

To assess pipeline performance, we analyzed the number of tumor confirmed mutations (considered as true positives for sensitivity assessment) and tumor unconfirmed mutations (which mostly contain false positives and serve as a proxy for specificity). Across the full dataset of 32 tumor samples, we identified 161 somatic mutations in tumor tissue (Additional file 1: Table S9), of which 104 were detected as initial candidates in plasma after UMI collapsing (Fig. 5C). After all filtering steps, the number of tumor-confirmed mutations decreased to 56, indicating that sensitivity is reduced by 46% due to error suppression. However, at the same time, tumor-unconfirmed mutations are drastically reduced by 99.99% from 433,464 to only 34, indicating a drastic increase in specificity that underlines the power of error suppression and filtering in the pipeline. To further understand whether the remaining 34 tumor-unconfirmed mutations contain additional true mutations or are errors, we investigated their cfDNA fragment distribution, since tumor derived DNA fragments are shorter, true mutations should show a shorter fragment profile [45–47]. Indeed, we observed a shift in fragment size for the 34 tumor-unconfirmed mutations, similar to the 56 tumor-confirmed mutations compared to healthy control plasma, indicating that these are enriched for tumor derived cfDNA fragments and contain additional true mutation calls (Fig. 5D). Of note, for the two matched samples derived from the same CRC patient at different time points, 6 out of 9 confirmed and tumor- unconfirmed mutations overlapped, demonstrating reproducible mutation calls.

As another performance evaluation, we compared mutation calls from tumor and healthy donor samples (as a negative control) for two versions of the pipeline: (1) an approach that incorporates patient matched PBMCs and analyzes all regions covered by the panel; and (2) a PBMC independent approach restricting these analysis to known hotspot mutations that are more likely to have somatic mutations, while excluding non-hotspot regions. In the absence of patient matched PBMC data, the focus on hotspot mutations should help to distinguish between somatic and germline mutations or clonal hematopoiesis. As expected, the pipeline with matched PBMC data detects more mutations in the CRC samples (64 versus 26) and fewer in healthy donors (2 versus 1), indicating improved sensitivity and specificity (Fig. 5E). Next, we compared our pipeline to Roche Avenio (Surveillance panel) on the independent test set of 10 CRC and 10 healthy plasma samples. Here we observe that the higher specificity of DEEPctMUT enables confident mutation detection in the VAF range of 0.03% − 0.5% VAF (Fig. 5F). Within this range, we observed no false mutation calls in healthy donors by DEEPctMUT, and only a single false call with DEEPctMUT without PBMC. In contrast, the Avenio assay produced nine false-positive mutation calls in healthy donors within the same VAF range. Of note although the Avenio assay has in principle the advantage of INDEL and fusion gene detection, none was identified in the CRC test set.

Next, we compared patient-level performance across pipelines. For Avenio, we considered mutations with VAF > 0.5% as informative (according to the manufacturer’s recommendation). For DEEPctMUT, we determined the informative threshold based on the lowest VAF achieving 95% patient-level specificity in cross-validation, which was VAF > 0.03% for DEEPctMUT and VAF > 0.05% for DEEPctMUT without PBMC (Additional file 2: Fig. S5A). In the cross-validation cohort, DEEPctMUT achieved 82% sensitivity and 96% specificity, whereas the version without PBMC filtering yielded 73% sensitivity and 92% specificity (Fig. 5G). In the independent test set, both DEEPctMUT versions showed 100% specificity, similar to Avenio. However, in terms of sensitivity, DEEPctMUT reached 100%, while DEEPctMUT without PBMCs achieved 90%, whereas Avenio detected mutations in only 50% of CRC patients, highlighting a substantial sensitivity improvement of up to 50%.

Since we had trained a more flexible panel-independent variation of DeepES (PI DeepES) that allows application for other ctDNA detection panels, we tested whether PI DeepES can improve prediction from the existing Avenio data. Therefore, we applied PI DeepES and RF directly on the BAM files generated by the Avenio UMI-collapsing pipeline, instead of the Avenio native processing pipeline. Interestingly, our approach was able to improve prediction performance relative to the native Avenio pipeline, increasing sensitivity to 80%, although specificity decreased to 90%, resulting in an overall 10% gain in accuracy, highlighting the generalization of the error suppression model in PI DeepES (Fig. 5F and G).

Furthermore, we analyzed whether mutational burden, as estimated by number of tumor mutations on TRON CRC panel, impacts detection sensitivity. Interestingly, we did not observe differences in mutational burden between CRC negative and positive samples (Additional file 2: Fig. S5B). When patients were categorized into high-burden ($\:\ge\:$ 4 mutations) and low-burden ($\:<$ 4 mutations) groups, no significant difference in sensitivity was observed (Additional file 2: Fig. S5C). When we investigated clinical characteristics, we observed a higher number of mutations and higher VAF levels in patients with metastatic cancer (Wilcoxon rank-sum test, *p* value = 0.026; Fig. 5H and Additional file 2: Fig. S5D). Furthermore, advanced stage cancers (III and IV) displayed both a greater number of mutations and mutations with higher VAFs compared to earlier stage cancers (Additional file 2: Fig. S5E and F).

Of note, also in early stages (I and II) that are often particularly challenging to detect due to low ctDNA levels, we identified in 62.5% patients as ctDNA positive, indicating high sensitivity across a wide range of patients. 

## Discussion

Unlike tumor-informed ctDNA detection assays, tumor-naïve approaches do not require prior analysis of a patient’s tumor sample. However, this versatility comes at the cost of reduced sensitivity for low–VAF mutations, because distinguishing true low–VAF variants from sequencing errors is challenging without prior knowledge of patient-specific mutations. Due to performance limitations of current error-correction techniques, these assays typically either target only a few hotspot mutations that are highly recurrent in the respective cancer type or require ultra-deep sequencing of large DNA volumes with duplex consensus-based UMI collapsing to confidently call mutations. This approach is further constrained by the limited number of cfDNA molecules that can be obtained from a small plasma sample. Both strategies suffer from reduced sensitivity: the former fails to detect rare or unique patient-specific mutations, which make up the majority of somatic variants [48, 49], while the latter is limited by the inefficiency of duplex consensus collapsing, which yields a limited number of usable ctDNA fragments [50, 51]. 

Improvement of error polishing techniques depends on the availability of high-quality ground truth data consisting of plasma samples with confirmed positive and negative mutations, which is challenging to obtain. Here, we generated and made available extensive ground truth data with different strategies. Our cell line dataset provides 6204 positive mutation datapoints across four VAF levels ranging from extremely low 0.00005% to 10%, providing an even wider VAF range as the previously published reference benchmarking dataset from the FDA-led Sequencing Quality Control Phase 2 (SEQC2) project (40.000 positive mutation data points across four VAF levels from 0.0625% to 5%) [42, 52, 53]. Thus, we offer more data points at the challenging and clinically highly relevant VAF range below 0.1%. Furthermore, we characterized 50 healthy plasma and 32 CRC plasma samples with 161 tumor-confirmed mutations. For comparison, the TracerX ctDNA study provides 551 confirmed ctDNA mutations in 46 NSCLC samples and iDES was established on 30 healthy plasma samples [23, 54]. All samples were deeply characterized by 20.000X sequencing, thus we provide a substantial amount of novel ground truth data across a wide variety of samples. 

Based on our data, we developed three complementary error polishing steps (RF, DeepES and matched-PBMC) that enable accurate identification of mutations as low as 0.03% VAF. Detection of short insertions and deletions (indels), which are frequent in microsatellite instability-high tumors (15% of CRC cases), is currently not supported [55]. Integration of indels requires dedicated training, which is why we focused on substitutions that makeup the majority of somatic mutations in CRC and other cancers (> 95%) [56]. We directly compared our approach to the Roche Avenio Surveillance panel, which covers also indels and gene fusions, and utilizes iDES to achieve high sensitivity down to 0.5% VAF [57]. In an independent test cohort we observed nonetheless up to 50% higher patient-level sensitivity, also without the additional matched-PBMC filtering step. This improvement is primarily achieved from the enhanced specificity of DeepES, which achieved a fivefold lower error rate in healthy cfDNA compared to iDES, thereby enabling more accurate variant calls at lower VAFs. 

Our dataset contains a wide range of low to high-grade CRC pre-surgery plasma samples, with cfDNA total input amounts ranging from 6 ng to 100 ng. A comparative study of tumor-naïve ctDNA assays detect mutations robustly only at VAFs higher than 0.5%, only few approaches maintained high sensitivity at 0.125% VAF — and even then, only with sufficient DNA input (~ 30–50 ng) [58]. At 0.125% VAF and 10 ng input, sensitivity dropped sharply, while tumor-informed assays routinely detected recurrence in CRC at median VAF as low as 0.028% [59]. With DEEPctMUT, we achieved high sensitivity for stage IV (94%) which is maintained also at lower stage II and III CRC patients (88%) and drops to 50% for stage I, thus exceeding other commercial tumor-agnostic assays and is on par with tumor-informed or multi-modal assays that use epigenetic information [1, 60–63].

This increase in sensitivity is particularly relevant for clinical application, as in minimal residual disease (MRD) monitoring up to 80% of detected mutations are below the typical tumor-agnostic detection threshold of 0.1% and only 67% of recurrences that were identified by tumor-informed assays are detected with tumor-agnostic assays [64]. While tumor-informed assays maintain a performance advantage over our DEEPctMUT pipeline, we can increase performance, especially in the clinical relevant range below 0.1% VAF also in critical samples with low cfDNA input amounts. Thus we could improve sensitivity especially for CRC grade I and II pre-surgery samples that similarly to MRD samples have very low ctDNA levels around 0.1% [65]. We focused here on pre-surgery samples because these are – contrary to post surgery samples for MRD detection – ctDNA positive and have therefore advantages for ground truth data generation, which was a focus of the study. Nonetheless, it will be important to evaluate the DEEPctMUT pipeline on a large cohort addressing a clinical question such as MRD detection, which was beyond the scope of this study.

Compared to other existing error polishing approaches such as iDES, which are limited by the scarcity of healthy plasma samples needed to effectively model position and substitution specific background sequencing errors [23, 24], we addressed this challenge with a novel deep learning formulation in DeepES. This approach incorporates additional informative features—such as trinucleotide context and fragment size—thereby compensating limited available healthy plasma data. Moreover, since DeepES derives its labels directly from the sequencing data of healthy samples, it does not require manual labeling, allowing for the easy generation of a massive training dataset. This formulation also paves the way for incorporating more informative biological features and enables the use of more advanced learning methods (e.g., transformers or convolutional neural networks) in the future to further improve error polishing. Importantly, because error rates depend on multiple technical and biological features that deep learning can effectively capture, we showed that a panel-independent version of DeepES can be trained using only these features, without position- and substitution-specific VAFs from healthy plasma, and still achieve reasonable performance. Moreover, since the Random Forest and PBMC polishing steps are panel-independent, combining them with panel-independent DeepES enables adaptation to other tumor types without generating additional healthy plasma data. This makes DeepES and the entire DEEPctMUT detection pipeline a versatile polishing method that can be readily implemented into other ctDNA detection methods, reducing both time and cost for development.

## Conclusions

In this paper, we developed DEEPctMUT, a novel pipeline that combines multiple error polishing steps, including an innovative feature-rich deep learning approach for accurate tumor-naïve ctDNA detection pipeline. We provide an extensive ground truth dataset for different types of errors that were used for training and testing and can support further development of error polishing methods. The pipeline itself is available on GitHub under open source license. Due to its versatility, DEEPctMT can be easily adapted to other sequencing panels without the need to generate additional ground truth data. Thus, our pipeline may pave the way for versatile ctDNA detection for multiple clinical questions. 

## Supplementary Information

Additional file 1. Supplementary Tables (Table S1-S9).

Additional file 2. Supplementary Figures (Fig. S1-S5).

## Data Availability

All sequencing data is available within European Nucleic Archive (ENA) under the accession number: PRJEB113702 [66]. The trained models and the DEEPctMUT pipeline are available under open access licence in GitHub https://github.com/TRON-Bioinformatics/DEEPctMUT [67].

## Abbreviations

ctDNA: Circulating tumor DNA
cfDNA: Cell-free DNA
CRC: Colorectal cancer
UMI: Unique molecular identifier
VAF: Variant allele frequency
MRD: Minimal residual disease
PPM: Parts per million
iDES: Integrated digital error suppression
DeepES: Deep error suppression
SEQC2: Sequencing quality control phase II
RF: Random forest
PI DeepES: Panel-independent DeepES
CNN: Convolutional neural networks
AUC: Area under the curve
indels: Insertions and deletions
SNV: Single nucleotide polymorphism
PBMC: Peripheral blood mononuclear cell
AGL: Agilent Custom Comprehensive Cancer Panel v2
BRP: Burning Rock DX OncoScreen Plus
IDT: Integrated DNA Technologies xGen Pan-Cancer Panel
IGT: iGeneTech AIOnco-seq
ILM: Illumina TruSight Tumor 170
QGN: QIAGEN Comprehensive cancer panel
ROC: Roche SeqCap EZ Choice custom PHC Panel
TFS: Thermo Fisher Oncomine Comprehensive Assay v3
