# Supplementary material for: Tumor-naïve ctDNA detection with deep learning-enhanced error suppression for sensitive mutation calling
Source: Genome Med. 2026 Jun 23;18:90. doi: 10.1186/s13073-026-01694-y (PMC13289477; doi:10.1186/s13073-026-01694-y)
Supplement: Supplementary file 2 — Additional file 2. Supplementary Figures (Fig. S1-S5). [file 13073_2026_1694_MOESM2_ESM.docx]

# Supplementary Figures


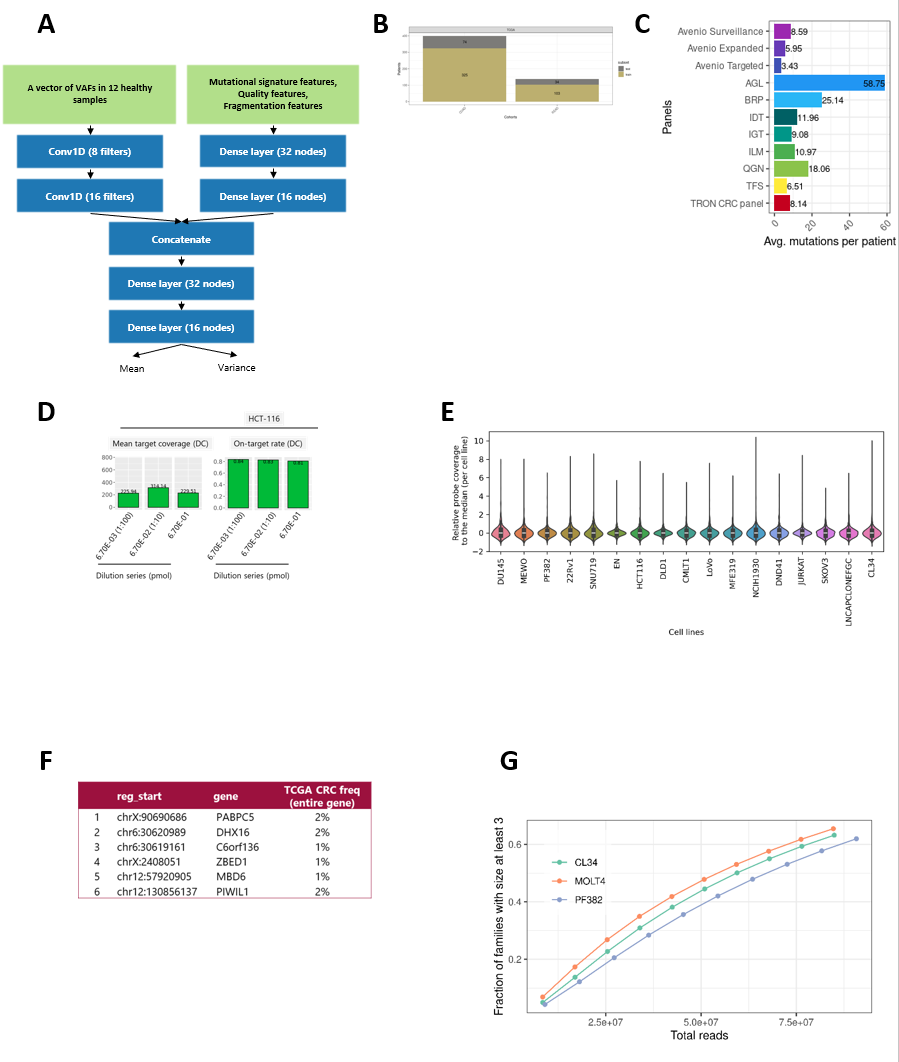


Fig S1: DeepES architecture and Design of CRC capture panel

(A) Schematic showing the architecture of DeepES. (B) The panel was designed based on TCGA COAD and READ cohorts, which we separated in training (green) and test (grey) cohorts as indicated. (C) Shown are the average number of mutations according to test cohort (n=108) for TRON CRC and other ctDNA panels. (D) Using IDT xGEN custom capture panel, mean target rate and on target rate are stable across a wide range of probe concentrations (undiluted HCT-116 cell line, 1:10 and 1:100 over the manufacturer’s recommendation). (E) Relative probe raw sequencing coverage in cell line samples for 834 probe regions. (F) List of six regions that are systematically underrepresented, indicated is also the gene name at the locus and the genes mutation frequency in TCGA [COAD + READ]cohorts. (G) Fraction of read families with at least three reads according to total raw read coverage across three exemplarily pure cell line samples.


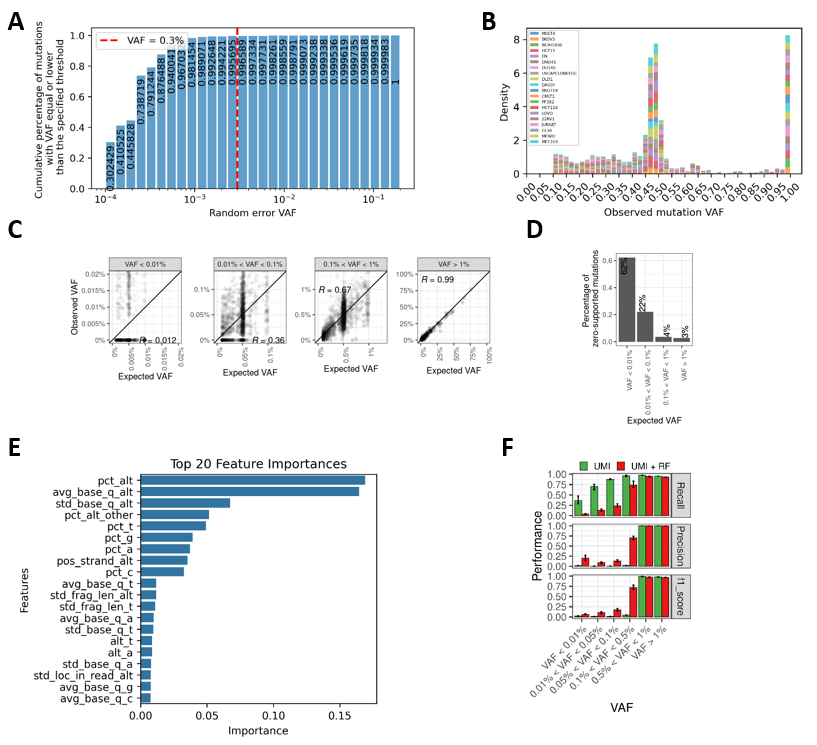


Fig S2: Characterization of random error and true mutations on pure cell lines, performance of sequencing on diluted cell lines, and features importance for the RF model

(A) Cumulative distribution of error rate of randomly selected positions in the panel. 99% of the errors have VAF < 0.3% (indicated by the dashed line). (B) Density plot showing the number of mutations with VAF > 10% per cell line across VAF ranges. (C) Correlation plot for expected VAF and observed VAF after dilution sequencing. (D) The number of expected mutations in each VAF range is shown. (E) Feature importance of the RF model. pct_alt: VAF of the alternate allele, avg_base_q_alt: Average base quality of bases supporting the alternate allele, std_base_q_alt: Standard deviation of bases supporting the alternate allele, pct_alt_other: Reads supporting alternate base / Reads supporting other non-reference bases, pct_t: VAF of base T, pct_g: VAF of base G, pct_a: VAF of base A, pos_strand_alt: Percentage of reads on positive strand, supporting the alternate allele, pct_c: VAF of base C, avg_base_q_t: Average base quality of T bases, std_frag_len_alt: Standard deviation of template length of reads supporting the alternate allele (using pysam template_length), std_frag_len_t: Standard deviation of template length of reads supporting base T (using pysam template_length), avg_base_q_a: Average base quality of bases supporting the alternate allele*, std_base_q_t: Standard deviation of bases supporting the base T, alt_t: Equals 1 if alternate base is T, alt_a: Equals 1 if alternate base is A, std_base_q_a: Standard deviation of bases supporting the base A, std_loc_in_read_alt: Standard deviation of positions of the bases within the reads supporting the alternate allele, avg_base_q_g: Average base quality of G bases, avg_base_q_c: Average base quality of C bases*. (F) Mutation detection performance (recall, precision, and f1) for UMI correction (green) and UMI correction with RF model (red) across different VAF ranges. The bars show the mean value while the error bars indicate the maximum and minimum values across 3 replicates.


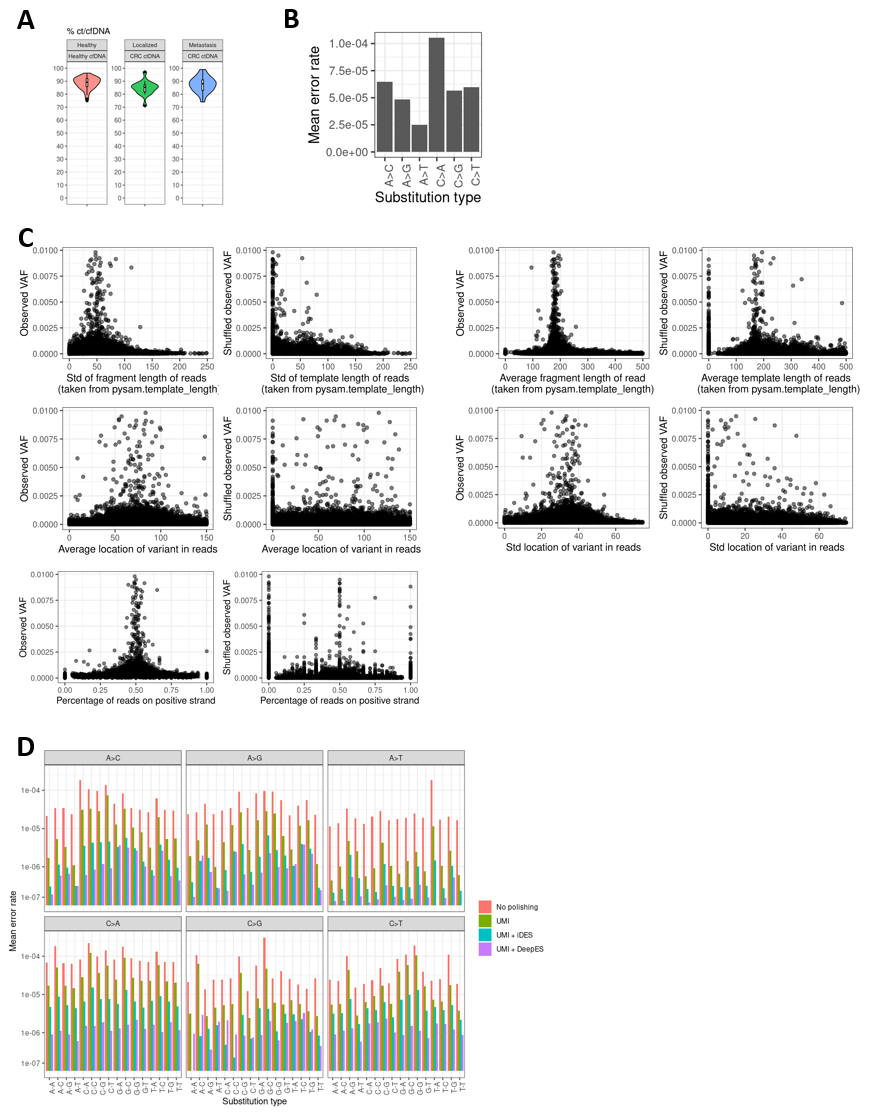


Fig S3: DeepES uses a wide range of features to polish errors

(A) Fractions of low molecular weight DNA across healthy and CRC patient samples. (B) Different substitutions have different error rate: this is a feature that gives us information about error rate (C) Other features that help DeepES predict the expected error rate. The subfigures on the right show the pattern of features if error VAF was assigned randomly to features. (D) When evaluated on 27 healthy plasma samples, trinucleotide-specific error rate after polishing a In,with DeepES hasa lower rate compared to iDES on most cases.


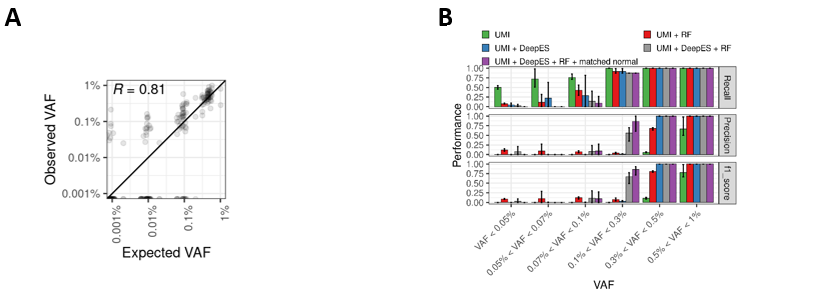


Fig S4: TWIST ground truth dataset

(A) Observed VAF after sequencing for all dilutions (1:10, 1:50, 1:500, 1:5000) is shown against the calculated expected VAF. R indicates the Pearson coefficient. (B) Recall, precision, and f1- score are shown for UMI (green), UMI + DeepES (blue), UMI + RF (red), UMI + DeepES + RF (grey), and UMI + DeepES + RF + matched PBMC (pink) across different VAF ranges. The bars show the mean value while the error bars indicate the maximum and minimum values across 3 replicates.


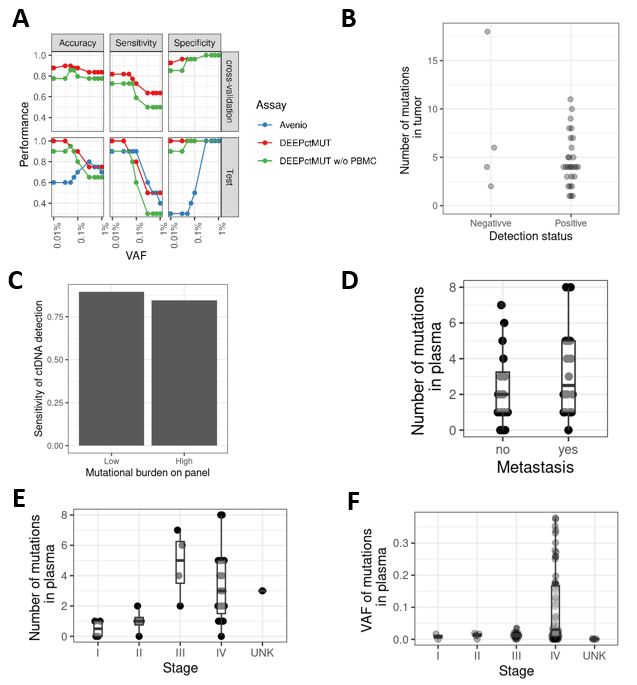


Fig S5: ctDNA detection in patient samples and correlation with clinical features

(A) Patient-level sensitivity, specificity, and accuracy of Avenio, DEEPctMUT, and DEEPctMUT without PBMC assays for different minimum VAF thresholds to determine a patient as ctDNA positive is specified. (B) Number of tumor mutations on panel in ctDNA positive and negative patients. (C) High mutational burden patients are defined by having ≥4 mutations in the tumor sample on panel. Low mutational burden are defined by having <4 mutations. (D, E) Average number of mutations according relapse and metastasis and stage for detection with the full pipeline utilizing RF, DeepES and PBMCs. (F) VAF of mutations in plasma according stage as detected with the full pipeline utilizing RF, DeepES and PBMCs.
